# Draft genome sequence of *Sphingomonas paucimobilis* strain Sph5, isolated from tap water filtration membrane

**DOI:** 10.1128/MRA.00345-23

**Published:** 2023-12-01

**Authors:** Sara Koroli, Kristina Buss, Joy M. Blain, Gautam Sai Nakka, Mina Hong, Robert JC McLean, Caroline M. Plugge, Jiseon Yang

**Affiliations:** 1 Biodesign Center for Fundamental and Applied Microbiomics, Biodesign Institute, Arizona State University, Tempe, Arizona, USA; 2 College of Medicine, University of Arizona, Phoenix, Arizona, USA; 3 Bioinformatics Core Facility, Biosciences, Knowledge Enterprise, Arizona State University, Tempe, Arizona, USA; 4 Genomics Core Facility, Biosciences, Knowledge Enterprise, Arizona State University, Tempe, Arizona, USA; 5 Department of Biology, Texas State University, San Marcos, Texas, USA; 6 Wetsus, European Centre of Excellence for Sustainable Water Technology, Leeuwarden, the Netherlands; University of Delaware College of Engineering, Newark, Delaware, USA

**Keywords:** draft genome, WGS, water, *Sphingomonas*

## Abstract

Sphingomonadaceae are common membrane colonizers and biofilm formers. As part of our studies on long-term genetic changes in drinking water biofilm species, we report the draft genome sequence of *Sphingomonas* strain Sph5, isolated from a tap water filtration membrane. The isolate was determined as *Sphingomonas paucimobilis* through whole genome sequencing and *de novo* assembly.

## ANNOUNCEMENT

Widely used for drinking water, membrane filtration systems face biofouling due to microbe accumulation and biofilm formation. Studies reveal Sphingomonadaceae as common initial colonizers, persisting dominantly during biofilm growth (1 – 4). Here, we report the genome sequence of a prevalent isolate.

*Sphingomonas* spp. Sph5 was isolated from a biofilm on a Nadir MP005 microfiltration membrane used for drinking water biofouling studies (1, 5). Sph5 was previously reported to be phylogenetically closest to *Sphingomonas sanguinis* strains BAB-7166 (99%) and NBRC 13937 (99%) based on 16S rRNA sequencing and cultivation methods (1). However, the whole genome sequence (WGS) was not reported. WGS analysis is critical to uncover strain-specific traits associated with membrane biofouling. Here, we report the WGS of *Sphingomonas* spp. Sph5 using Illumina short-read sequencing and *de novo* assembly methods.

We received the *Sphingomonas* spp. Sph5 water isolate from Wetsus, Netherlands. Single isolated colonies were obtained on Reasoner’s 2A agar (R2A, Teknova R0005; Difco 214530) and cultured in R2A broth at room temperature. Genomic DNA was extracted using DNeasy UltraClean microbial kit (Qiagen 12224) following the company-provided protocol.

Sequencing libraries were generated using Kapa’s Hyperplus kit (KK8514) and IDT adapters (#00989130v2). Quality was verified using an Agilent Tapestation and qPCR (NEBNext Library Quant Kit, E7630L) on Thermo Fisher’s Quantstudio5.

Nineteen million 2 × 150 bp reads were obtained on a NovaSeq (Illumina) at Anschutz Medical Campus Genomics and Microarray Core, with an average Phred score of 33–36 calculated by FastQC [v0.11.9 (6)].

Assembly was performed with SPADES, default isolate settings [v3.15.2 (7)]. Contigs were aligned to NCBI databases with DIAMOND [v2.0.13 (8)]. Completeness was estimated using BUSCO [v5.2.2 (9)] proteobacteria marker genes. Of the 219 genes, there were 214 (97.7%) complete and single copy, 2 (0.9%) duplicated, 1 (0.5%) fragmented, and 2 (0.9%) missing.

For species identification, average nucleotide identity (ANI) was calculated between all complete *Sphingomonas* genomes and Sph5 with PYANI [v0.2.11 (10)] and MUMMER [v4.0.0 (11)]. ANI scores between Sph5 and *S. paucimobilis* ranged from 99.77%–99.95%, while ANI between Sph5 and *S. sanguinis* was 87.74%.

Annotations were predicted with Prokka [v1.14.6 (12)], given *S. sanguinis* and *S. paucimobilis* references (Table 1), and used for orthogroup-based phylogeny construction with Orthofinder [v2.5.4 (13)], MAFFT [v7.490 (14)], and FastTree [v.2.1.10 (15)].

**TABLE 1:** Summary of the draft whole genome sequences of *Sphingomonas paucimobilis* Sph5 from the tap water filtration membrane

Variable	Data
Genus and species	*Sphingomonas paucimobilis*
Strain	*Sph5*
NCBI accession no.	JAMRJL000000000
Country	The Netherlands
Source	Biofilm on Nadir MP005 microfiltration membrane
Feed water source	Tap water from the city of Leeuwarden
Genome size (bp)	4680620
N_50_ (bp)	4016723
Scaffold reference	ASM1602843v1
Scaffolds ≥50,000 bp	29, 4
Mean coverage	1,089×
G + C content (%)	65.30
No. of annotated CDS	3,342
No. of raw reads	19,032,602
No. of reads used for assembly	19,032,602
No. of coding sequences	4,321

**Fig 1:**
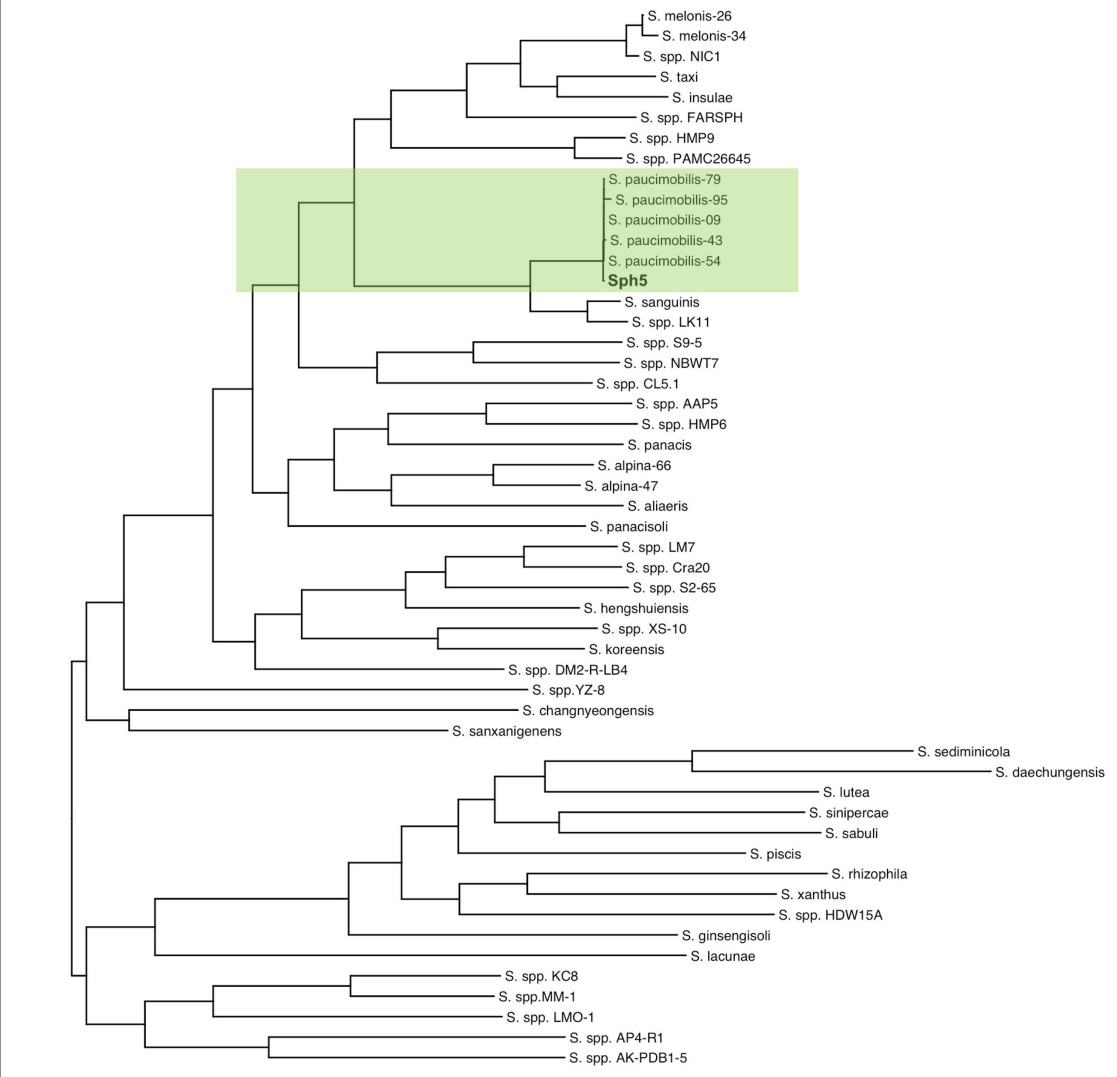
Orthofinder-generated rooted tree of 50 complete *Sphingomonas* genomes with the Sph5 strain. Branch lengths represent evolutionary distance. We used the multiple-sequence alignment option with MAFFT and used FastTree to infer the trees. The mathematical parameters used are built in to the OrthoFinder tool, and we did not alter them from the default.

The WGS phylogeny (Fig. 1) confirms that Sph5 is closest to *S. paucimobilis* while related to *S. sanguinis*. As a result, *S. paucimobilis* reference genomes were used for RagTag [v2.1.0 (16)] scaffolding of Sph5 contigs. ASM1602843v1 was the best scaffold (93.6% total length in three longest contigs of resulting assembly). Realignment of raw reads to ASM1602843v1-scaffolded Sph5 assembly with BWA [v0.7.17 (17)], SamTools [v1.12 (18)], and Picard [v2.25.0 (19)], evaluated by Mosdepth [v0.3.3 (20)], supports this (99.48% reads aligned, 1,089× coverage).

Re-annotation with Prokka predicts 4,321 CDS, 3 rRNAs, 54 tRNAs, 1 tmRNA, and 1 repeat region. Of the CDS, 3,342 could be functionally annotated.

## Data Availability

The assembly and raw data are deposited at NCBI through the SRA genome submission portal under the accession numbers JAMRJL000000000 and SRR25337554. The version deposited in this paper is the first version.
